# Traditional Chinese medicine for the prevention and treatment of COVID-19: A protocol for systematic review and network meta-analysis

**DOI:** 10.1097/MD.0000000000028375

**Published:** 2021-12-30

**Authors:** Xin Wang, Yafeng Wang, Hairu Lu, Luqing Yan

**Affiliations:** Qinghai Provincial People's Hospital, Xining, Qinghai, China.

**Keywords:** COVID-19, network meta-analysis, traditional Chinese medicine

## Abstract

**Background:**

The coronavirus disease 2019 (COVID-19) pandemic spread to most of the world's countries during its first and subsequent waves, often increasing in an almost exponential trend. Traditional Chinese medicine has played a vital role in this epidemic. Nonetheless, size of effect, certainty of the evidence, optimal therapy regimen, and selection of patients who are likely to benefit most are factors that remain to be evaluated. This study aims to assess and rank where appropriate the relative effects of interventions for the prevention and treatment of COVID-19.

**Method:**

This study will follow the Preferred Reporting Items for Systematic Reviews and Meta-analysis Protocols. We will search Chinese electronic database (CBM, Wanfang and CNKI) and international electronic databases (PubMed, Embase, Cochrane Library, and Web of Science) for identify all relevant published studies. Study selection, data collection and assessment of study bias will be conducted independently by a pair of independent reviewers. The Cochrane risk of bias tool will be used for the risk of bias assessment. We will use the advance of GRADE to rate the certainty of network meta-analysis. Data analysis will be performed with R-3.6.1 and WinBUGS software.

**Results:**

The results of this study will be published in a peer-reviewed journal.

**Conclusion:**

This systematic review and network meta-analysis will use both direct and indirect evidence to compare the differences of all Traditional Chinese medicine treatment for COVID-19 patients, providing decision-makers and clinical practitioners with a complete, high-quality and up-to-date synthesis of evidence.

## 1 Introduction

Coronavirus disease 2019 (COVID-19) is a rapidly spreading infectious disease caused by severe acute respiratory syndrome coronavirus 2 (SARS-CoV-2). SARS-CoV-2 is a novel coronavirus first documented in December 2019 in an outbreak in Wuhan, Hubei, China.^[1]^ Over the first six weeks of the new decade, SARS-CoV-2 transmission spread from China to several other countries. On 11 March 2020, the World Health Organization (WHO) declared the current COVID-19 outbreak a pandemic.

COVID-19 can cause various clinical manifestations, from non-specific flu-like symptoms (fever, dry cough, fatigue) to severe hypoxaemia, multi-organ failure, and death.[^2^,^3^,^4^] The median incubation time is estimated to be 5 to 6 days, and 97.5% of symptomatic cases develop symptoms within 11.5 days of exposure. The most commonly reported symptoms incudeds sore throat, cough, fever, headache, fatigue, and myalgia or arthralgia. Other symptoms include dyspnoea, chills, nausea or vomiting, diarrhoea, and nasal congestion. Despite intensive international efforts to contain its spread, it has resulted in more than 250 million confirmed cases and more than 5 million deaths worldwide until November 2021. Given the severity of the COVID-19 pandemic and the scarcity of effective treatments, there is an urgent need for effective treatments to save lives and relieve the heavy burden on health systems, especially as the virus continues to evolve, with the potential to increase transmission capacity and limited global vaccine supplies.

Traditional Chinese medicine (TCM) has a long history and played an indispensable role in the prevention and treatment of several epidemic diseases and plagues. During the epidemic period of SARS in 2003 and H1N1 influenza,^[5]^ TCM played a vital role in fighting the epidemic, the Chian's National Health Commission (NHC) has recommended TCM as a strategy for COVID-19 treatment.^[6]^ Treating patients as an organic whole is the prerequisite for TCM to treat all diseases. COVID-19 is primarily located in the lungs, the pathogenesis of COVID-19 is mainly “wet, heat, poison, stasis, deficiency” and the lesions are mainly in the spleen, lungs and stomach.^[7]^ From the mechanism of TCM, we believe that lungs are delicate, so the disease first affects lungs’ function.^[8]^ Based on current clinical results, TCM has displayed some efficacy in combating COVID-19. In particular, 3 TCM prescriptions-Qingfei Paidu decoction, Huashi Baidu decoction, and Xuanfei Baidu decoction have been proven effective in clinical.^[9]^ COVID-19 patients taking Jinhua Qinggan granules recovered faster than patients who did not take the granules, testing negative for coronavirus more than 2 days sooner.^[10]^ Moreover, the combination of TCM and Western medicine reduced adverse events and other complications induced by glucocorticoid, anti-biotic and anti-viral treatment.^[11]^ Also, several studies have shown that none of the medical staff was infected in the TCM hospitals or when using TCM methods.^[12]^ The 4-step TCM method of defense, qi, nutrient, and blood explains why no case progressed from mild to severe in the treatment of COVID-19 by TCM.^[13]^

Considering that several clinical practice results showed that TCM plays significant role in the treatment of COVID-19, bringing new hope for the prevention and control of COVID-19. Our study will perform a comprehensive systematic review and network analysis to assess and rank where appropriate the relative effects of TCM interventions for the treatment of COVID-19.

## 2 Methods

### 2.1 Study registration

This protocol will be reported according to preferred reporting items for systematic review and meta-analysis protocols (PRISMA-P)^[14]^ and this network meta-analysis will be conducted and reported according to PRISMA Extension version (PRISMA-NMA).^[15]^ This protocol has been registered on the open Science framework (OSF) (OSF DOI: 10.17605/OSF.IO/XRMDA).

### 2.2 Search strategy

We will search the following six databases: PubMed, Embase.com, Cochrane Library, CNKI (China National Knowledge Infrastructure), WanFang and CBM (Chinese Biomedical Database). We will also search several resources for unpublished and ongoing studies. The search terms and basic search strategy were as follows: (2019-nCoV OR SARS-CoV-2 OR Novel coronavirus OR COVID-19 OR coronavirus) AND (traditional Chinese herbal medicine OR TCM or traditional Chinese medicine) AND random∗. The references of relevant systematic reviews/meta-analyses will be tracked to identify additional studies. We will provide specific search strategy sample of PubMed and will be shown in Appendix 1.

### 2.3 Eligibility criteria

#### 2.3.1 Types of studies

We will include randomized controlled trials comparing TCM for COVID-19 patients, whatever the trial design, including cluster-randomized trials and crossover trials. We will exclude studies about prognosis, systematic reviews and meta-analyses and diagnostic test accuracy studies.

#### 2.3.2 Types of participants

We will include individuals with a confirmed diagnosis of COVID-19 and did not exclude any studies based on gender, ethnicity, disease severity, or setting.

#### 2.3.3 Types of interventions

We will include all available regimens of TCM that were evaluated in randomised trials. The control group could receive the standard of care/active intervention, placebo, or best supportive care.

#### 2.3.4 Types of outcome measures

We will base our outcome selection on the CORE outcome sets (COS) developed by the WHO. The primary outcome includes the (1) Incidence of serious adverse events; (2) WHO Clinical Progression Score level 6 or above (2) clinical efficacy (i.e., overall response rate, cure rate, hospital stay); (3) Clinical improvement (D7, D14, D28, D60, D90), defined as hospital discharge or improvement on the scale used by trialists to evaluate severity; (4) C. laboratory indicators (i.e., lymphocyte percentage, white blood cell count, C-reactive protein).

### 2.4 Study selection and data extraction

Two review authors (WYF and LHR) will independently screen the titles and abstracts according to the inclusion criteria. We will record the selection process insufficient detail to complete a PRISMA flow chart. Two review authors (LHR and YLQ) working independently will extract all data and will call upon a third review author to resolve disagreements. We will design and use a specific structured online data extraction form to ensure consistency of information. data extracted will include study characteristics (such as first author, publication year, country, setting and journal), number of participants randomized, patient characteristics (sampling, age and gender), intervention details, outcome measures.

### 2.5 Assessment of risk of bias in included studies

Two review authors (WYF and WX) will assess each study with the Cochrane ’Risk of bias 2’ (RoB 2) tool^[16]^ for randomized controlled trials. The Cochrane RoB 2 tool is structured into 5 domains: (1) risk of bias arising from the randomization process; (2) risk of bias due to deviations from intended interventions; (3) risk of bias due to missing outcome data; (4) risk of bias in measurement of the outcome; and (5) risk of bias in selection of the reported result. We will use a threshold of >20% missing data as indicative of high risk of bias for missing data. Any disagreements between reviewers were resolved by discussion or consultation with a third reviewer.

### 2.6 Data synthesis and analysis

For dichotomous outcomes, we will use as measure of effect the risk ratio accompanied by the 95% CI. For continuous and time-to-event outcomes we will use the standardized mean difference, or mean difference, if all studies use the same scale; and the HR, respectively. In the pairwise meta-analysis, different comparisons from multi-arm trials will be analyzed separately. In the network meta-analyses, we will properly account for the inherent correlation in multi-arm trials.

We will present the data by pairwise comparison and network diagrams with nodes representing the interventions being compared and lines representing the available direct comparisons in the studies.

### 2.7 Dealing with missing data and Assessment of heterogeneity

For missing outcome data, we will extract the number of participants who dropped out before the completion of the study and describe how missing outcome data were handled by the study authors. In addition, to assess the potential impact of missing outcome data on the results, we will conduct sensitivity analyses, making different assumptions. We will also conduct subgroup analyses and network meta-regression analyses to explore statistical heterogeneity across trials and inconsistency between direct and indirect evidence. We will focus on following possible effect modifiers: gender, age, and race.

### 2.8 Assessment of the certainty of the evidence

We will use the GRADE approach^[17]^ to evaluate the confidence in the results of the pairwise comparisons for the critical outcomes and classify evidence as high, moderate, low, or very low certainty. To evaluate the confidence in the NMA for the critical outcomes, we will use the CINeMA tool^[18]^ that considers the following domains: within-study bias, across-studies bias, indirectness, imprecision, heterogeneity and incoherence. For within-study bias and indirectness, CINeMA calculates the contribution of each study in each network estimate and combines these contributions with the study-specific evaluations (low, moderate, high) to rate the relative effect for each comparison in the network.

## 3 Discussion

There is a clear and urgent need for more evidence-based information to guide clinical decision-making for COVID-19 patients. Whilst early clinical trials seemed to reproduce positive effects of TCM on clinical improvement, leading to widespread authorisation of emergency use, the currently available data are conflicting and uncertain. There is a need for a thorough understanding and an extensive review of the current body of evidence regarding the use of TCM for the treatment of COVID-19. This systematic review and network meta-analysis are to provide practicing clinicians, healthcare providers, and interested laypersons with reliable and evidence-based information that will lead to improvement in the treatment of COVID-19. And hope to fill current gaps by identifying, describing, evaluating, and synthesizing all evidence for TCM on clinical outcomes in COVID-19.

## Author contributions

**Conceptualization:** Xin wang.

**Data curation:** Yafeng Wang.

**Investigation:** Yafeng Wang.

**Project administration:** Xin wang.

**Resources:** Yafeng Wang, Luqing Yan.

**Software:** Yafeng Wang, Hairu Lu.

**Supervision:** Hairu Lu.

**Visualization:** Hairu Lu, Luqing Yan.

**Writing – original draft:** Xin wang, Luqing Yan.

**Writing – review & editing:** Luqing Yan.

## Supplementary Material

Supplemental Digital Content

## References

[1] Chen N Zhou M Dong X. Epidemiological and clinical characteristics of 99 cases of 2019 novel coronavirus pneumonia in Wuhan, China: a descriptive study. Lancet (London, England) 2020;395:507–13. 10.1016/S0140-6736(20)30211-7 PMC7135076 32007143

[2] The Novel Coronavirus Pneumonia Emergency Response Epidemiology T. The epidemiological characteristics of an outbreak of 2019 novel coronavirus diseases (COVID-19)—China, 2020. China CDC Wkly 2020;2:113–22. 34594836 PMC8392929

[3] Karagiannidis C Mostert C Hentschker C. Case characteristics, resource use, and outcomes of 10 021 patients with COVID-19 admitted to 920 German hospitals: an observational study. Lancet Respir Med 2020;8:853–62. 32735842 10.1016/S2213-2600(20)30316-7 PMC7386882

[4] Guisado-Vasco P Valderas-Ortega S Carralón-González MM. Clinical characteristics and outcomes among hospitalized adults with severe COVID-19 admitted to a tertiary medical center and receiving antiviral, antimalarials, glucocorticoids, or immunomodulation with tocilizumab or cyclosporine: a retrospective observational study (COQUIMA cohort). EClinicalMedicine 2020;28:100591. 33078138 10.1016/j.eclinm.2020.100591 PMC7557296

[5] Luo Y Wang CZ Hesse-Fong J Lin JG Yuan CS. Application of Chinese medicine in acute and critical medical conditions. Am J Chin Med 2019;47:1223–35. 31505937 10.1142/S0192415X19500629

[6] China NHCotPsRo. National Health Commission of the People's Republic of China Diagnosis and treatment of pneumonia caused by the 2019 new coronavirus (2019-nCoV). http://www.nhc.gov.cn/wjw/xinx/xinxi.shtml.

[7] YG W WS Q JJ M. Clinical features and syndrome differentiation of novel coronavirus pneumonia in traditional Chinese medicine. J Tradit Chin Med 2020;61:281–5.

[8] Bai Z Li P Wen J. Inhibitory effects and mechanisms of the anti-covid-19 traditional Chinese prescription, Keguan-1, on acute lung injury. J Ethnopharmacol 2021;285:114838. 34788645 10.1016/j.jep.2021.114838 PMC8590745

[9] National Administration of Traditional Chinese Medicine. Practitioners of traditional Chinese medicine select a batch of formulations, as exemplified by “three existing TCMs and 3 new formulations ”, to treat COVID-19. http://www.satcm.gov.cn/xinxifabu/meitibaodao/.

[10] Liu J Wang J Liu X Shen H. The role of traditional Chinese medicine in COVID-19: theory, initial clinical evidence, potential mechanisms, and implications. Altern Ther Health Med 2021;27 (S1):210–27. 34653019

[11] Dai YJ Wan SY Gong SS Liu JC Li F Kou JP. Recent advances of traditional Chinese medicine on the prevention and treatment of COVID-19. Chin J Nat Med 2020;18:881–9. 33357718 10.1016/S1875-5364(20)60031-0 PMC7832371

[12] Xian NX Zhang Z Li N Liu N. Study on treatment from heart for severe patients based on etiology and pathogenesis transmission of corona virus disease. Tradit Chin Med 2019;38:20–4.

[13] Zhao Z Li Y Zhou L. Prevention and treatment of COVID-19 using traditional Chinese medicine: a review. Phytomedicine 2021;85:153308. 32843234 10.1016/j.phymed.2020.153308 PMC7439087

[14] Shamseer L Moher D Clarke M. Preferred reporting items for systematic review and meta-analysis protocols (PRISMA-P) 2015: elaboration and explanation. BMJ (Clin Res Ed) 2015;350:g7647. 10.1136/bmj.g7647 25555855

[15] Hutton B Salanti G Caldwell DM. The PRISMA extension statement for reporting of systematic reviews incorporating network meta-analyses of health care interventions: checklist and explanations. Ann Intern Med 2015;162:777–84. 26030634 10.7326/M14-2385

[16] Sterne JAC Savović J Page MJ. RoB 2: a revised tool for assessing risk of bias in randomised trials. BMJ (Clin Res Ed) 2019;366:l4898. 10.1136/bmj.l4898 31462531

[17] Guyatt G Oxman AD Akl EA. GRADE guidelines: 1. Introduction-GRADE evidence profiles and summary of findings tables. J Clin Epidemiol 2011;64:383–94. 21195583 10.1016/j.jclinepi.2010.04.026

[18] Nikolakopoulou A Higgins JPT Papakonstantinou T. CINeMA: an approach for assessing confidence in the results of a network meta-analysis. PLoS Med 2020;17:e1003082. 32243458 10.1371/journal.pmed.1003082 PMC7122720

