# Supplementary material for: Traditional Chinese medicine for the prevention and treatment of COVID-19: A protocol for systematic review and network meta-analysis
Source: Medicine (Baltimore). 2021 Dec 30;100(52):e28375. doi: 10.1097/MD.0000000000028375 (PMC8718199; doi:10.1097/MD.0000000000028375)
Supplement: Supplemental Digital Content [file medi-100-e28375-s001.docx]

**The specific search strategy will be (taking PubMed as an example):**

1. COVID-19 [mh]

2. (COVID19* OR SARS-CoV-2 OR SARS coronavirus 2 OR 2019 nCoV OR severe acute respiratory syndrome coronavirus 2 OR corona virus disease 2019)). ti,ab.

3. 1 OR 2

4. traditional Chinese medicine[mh]

5. (TCM OR traditional Chinese medicine OR traditional Chinese herbal medicine OR).ti,ab.

6. 4 OR 5

7. 3 AND 6

8. randomized controlled trial[mh]

9. randomized controlled trial.pt.

10. controlled clinical trial.pt.

11. randomized.ab.

12. placebo.ab.

13. clinical trials as topic.sh.

14. randomly.ab.

15. trial.ti

16. 8 or 9 or 10 or 11 or 12 or 13 or 14 or 15

17. 7 and 16
